# Supplementary material for: Roles of Human Liver Cytochrome P450 Enzymes in Tenatoprazole Metabolism
Source: Pharmaceutics. 2022 Dec 21;15(1):23. doi: 10.3390/pharmaceutics15010023 (PMC9863764; doi:10.3390/pharmaceutics15010023)
Supplement: Supplementary file 1 [file pharmaceutics-15-00023-s001.zip › pharmaceutics-1983470-supplementary.pdf]

Article

Roles of Human Liver Cytochrome P450 Enzymes in Tenatoprazole Metabolism

Thien-Kim Le [1,†], Young Jin Park [2,†], Gun Su Cha [3], Fikri A. R. Hardiyanti Oktavia [4], Dong Hyun Kim [2,*] and Chul-Ho Yun [1,*]

[1] School of Biological Sciences and Technology, Chonnam National University, 77 Yongbongro, Gwangju 61186, Republic of Korea
[2] Department of Pharmacology and Pharmacogenomics Research Center, Inje University, College of Medicine, Bokjiro 75, Busanjin-Gu, Busan 47392, Republic of Korea
[3] Namhae Garlic Research Institute, 2465-8 Namhaedaero, Gyeongsang-namdo 52430, Republic of Korea
[4] School of Biological Sciences and Biotechnology, Graduate School, Chonnam National University, 77 Yongbongro, Gwangju 61186, Republic of Korea
* Correspondence: dhkim@inje.ac.kr (D.H.K.); chyun@jnu.ac.kr (C.-H.Y)
† These authors contributed equally to this work.

Table S1. Characteristics of tenatoprazole sulfide metabolites of HLMs by LC-QTOF-MS.

Metabolite	Reaction type	Formula	Theorerical mass [M + H]+	Measured mass [M + H]+	Fragement ion (m/z)	Error (δ) ppm	RT (min)	Abs. intensity
**M1**	Dihydroxylation	$C_{16}H_{18}N_4O_4S$	363.1121	363.117	330, 198, 180, 151, 136	12.0	2.2	342,419
**M2**	S-Oxygenation O-Desmethylation	$C_{15}H_{16}N_4O_3S$	333.1016	333.104	300, 285, 198, 180, 151, 136	8.2	2.3	26,456
**M3**	S-Oxygenation Hydroxylation	$C_{16}H_{18}N_4O_4S$	363.1121	363.116	330, 311, 214, 180, 152	11.6	2.5	43,807
**M4**	Hydroxylation N-Oxygenation	$C_{16}H_{18}N_4O_4S$	363.1121	363.117	345, 312, 214, 196, 152	12.9	3.0	76,514
**M5**	S-Oxygenation Hydroxylation	$C_{16}H_{18}N_4O_4S$	363.1121	363.116	214, 196, 184, 167, 152	10.8	3.1	64,820
**M6**	S-Oxygenation Hydroxylation	$C_{16}H_{18}N_4O_4S$	363.1121	363.116	214, 196, 184, 167, 152	9.5	3.3	31,773
**P1**	Hydroxylation	$C_{16}H_{18}N_4O_3S$	347.1172	347.121	314, 182, 150, 136, 120	10.7	3.4	2,058,281
**P2**	Hydroxylation	$C_{16}H_{18}N_4O_3S$	347.1172	347.121	314, 198, 150, 138, 121	10.2	3.8	1,716,432
**Tenatoprazole**	S-Oxygenation	$C_{16}H_{18}N_4O_3S$	347.1172	347.1217	198, 180, 168, 151, 136	12.9	4.0	4,332,256
**M7**	S-Oxygenation Hydroxylation	$C_{16}H_{18}N_4O_4S$	363.1121	363.115	345, 313, 198, 182, 152	9.1	4.5	31,183
**M8**	S-Dioxygenation	$C_{16}H_{18}N_4O_4S$	363.1121	363.117	299, 214, 196, 150	12.9	5.5	76,769
**Tenatoprazole sulfide**	Parent	$C_{16}H_{18}N_4O_2S$	331.1223	331.124	298, 283, 182, 150, 136	4.2	5.3	6,080,753

Table S2. Characteristics of tenatoprazole metabolites of CYP2C19 by LC-QTOF-MS.

Metabolites	Description	Formula	Theorerical mass $[M+H]^+$	Measured mass $[M+H]^+$	Error (δ) ppm	RT (min)	Abs intensity
**M1**	Hydroxylation	$C_{16}H_{18}N_4O_4S$	363.112	363.115	7.9	2.2	258121
**M4**	Hydroxylation<br>*N*-Oxygenation<br>Desulfoxidation	$C_{16}H_{18}N_4O_4S$	363.112	363.116	10.9	3.0	1131370
**P1**	Desulfoxidation<br>Hydroxylation	$C_{16}H_{18}N_4O_3S$	347.117	347.122	12.9	3.4	1155253
**P2**	Desulfoxidation<br>Hydroxylation	$C_{16}H_{18}N_4O_3S$	347.117	347.121	11.7	3.8	2160271
**Tenatoprazole sulfide**	Desulfoxidation	$C_{16}H_{18}N_4O_2S$	331.122	331.124	6.6	5.3	3270918
**Tenatoprazole**	Parent	$C_{16}H_{18}N_4O_3S$	347.117	347.121	11.8	3.95	2416865

Table S3. Characteristics of tenatoprazole metabolites of CYP3A4 by LC-QTOF-MS.

Metabolites	Description	Formula	Theorerical mass $[M+H]^+$	Measured mass $[M+H]^+$	Error (δ) ppm	RT (min)	Abs intensity
**M1**	Hydroxylation	$C_{16}H_{18}N_4O_4S$	363.112	363.115	8.4	2.2	110868
**M4**	Hydroxylation N-Oxygenation Desulfoxidation	$C_{16}H_{18}N_4O_4S$	363.112	363.115	8.4	3.0	90502
**M6**	Hydroxylation	$C_{16}H_{18}N_4O_4S$	363.112	363.115	7.6	3.3	72625
**P1**	Desulfoxidation Hydroxylation	$C_{16}H_{18}N_4O_3S$	347.117	347.121	12.3	3.4	467330
**Tenatoprazole sulfide**	Tenatoprazole sulfide	$C_{16}H_{18}N_4O_2S$	331.122	331.124	6.6	5.3	3607768
**M8**	S-Oxygenation	$C_{16}H_{18}N_4O_4S$	363.112	363.115	9.1	5.5	1322043
**Tenatoprazole**	Parent	$C_{16}H_{18}N_4O_3S$	347.117	347.121	11.5	3.96	6255520

Table S4. ^{1}H NMR chemical shifts of 1'-*N*-oxy-5'-hydroxytenatoprazole sulfide

	**^{1}H(d), ppm (DMSO-d_6 at 25 °C)**
5-OCH_3	3.800
H-6	6.469 (d, 1H, J=8.4Hz)
H-7	7.672 (d, 1H, J=8.4Hz)
2'-CH_2	4.661 (s, 2H)
3'-CH_3	2.279 (s, 3H)
4'-OCH_3	3.764 (s, 3H)
5'-CH_2	4.534 (s, 2H)
H-6'	8.305 (s, 1H)

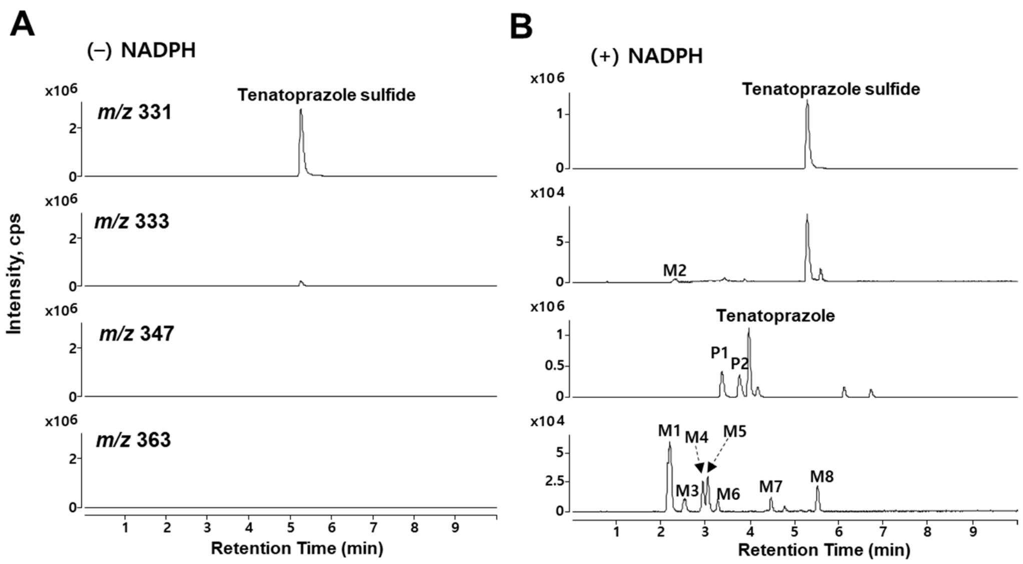

Figure S1. LC-QTOF-MS analyses of the metabolites of tenatoprazole sulfide oxidized by HLMs. The extracted ion chromatograms were constructed by incubating tenatoprazole sulfide with HLMs in the absence (**A**) and presence (**B**) of NADPH.

Figure S2. LC-QTOF-MS analyses of the products derived from the oxidation of tenatoprazole and tenatoprazole sulfide by HLMs. The MS spectra of the protonated molecular ions for tenatoprazole (**A**), tenatoprazole sulfide (**B**), and their metabolites (**C**-**K**) M1-M8, P1, and P2), shown Tables 1 and 2. An unidentified monohydroxylated product (peak 4 of Figure 2) is shown as M**5** at Figure S1h. Predicted chemical structure based on the LC-MS analyses were also shown.

(A)

Tenatoprazole
- m/z 347

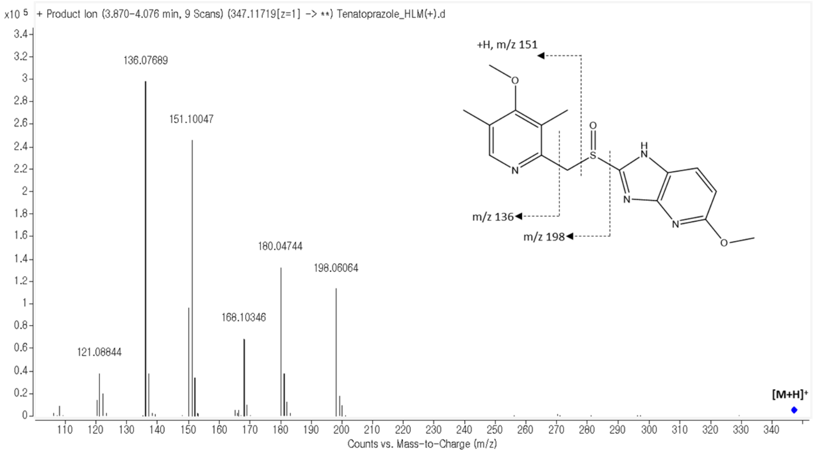

(B)

Tenatoprazole sulfide
- *m/z* 331

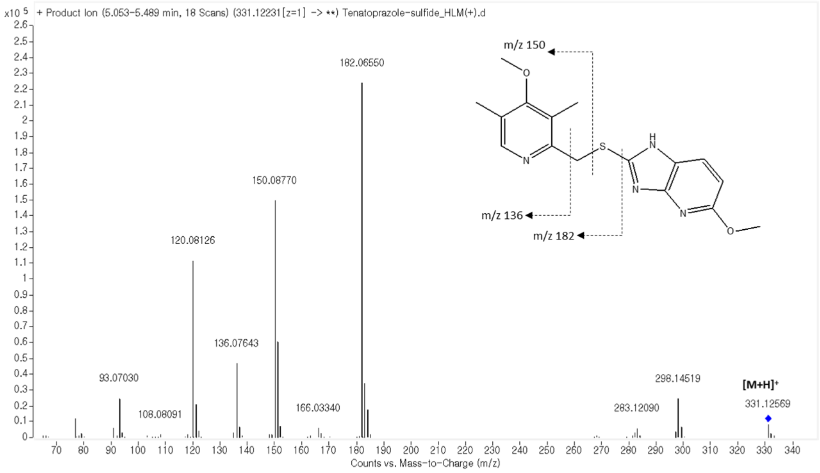

(C)

P1
- *m/z* 347

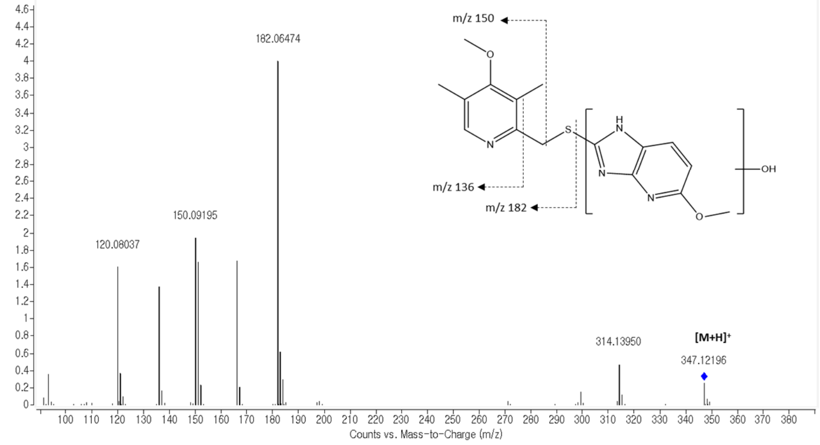

(D)

P2

- *m/z* 347

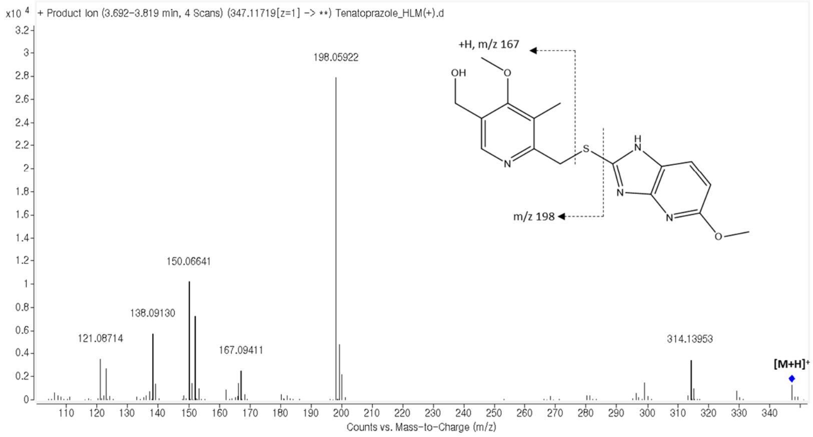

.

(E)

M1

- *m/z* 363

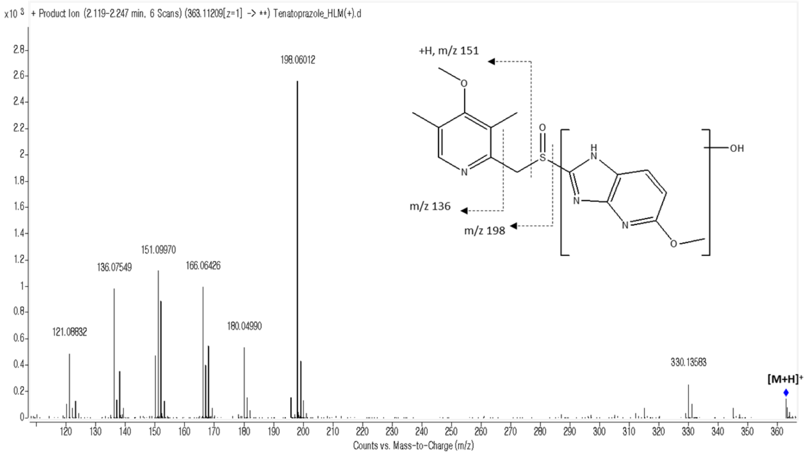

(F)

M2

- *m/z* 333

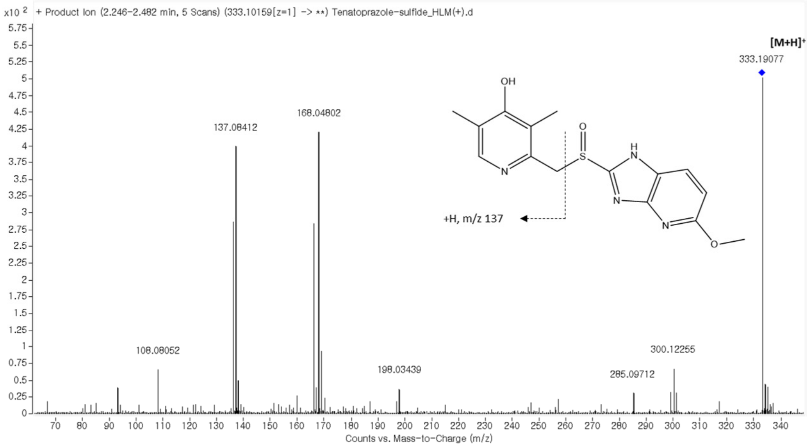

(G)

M3

- *m/z* 363

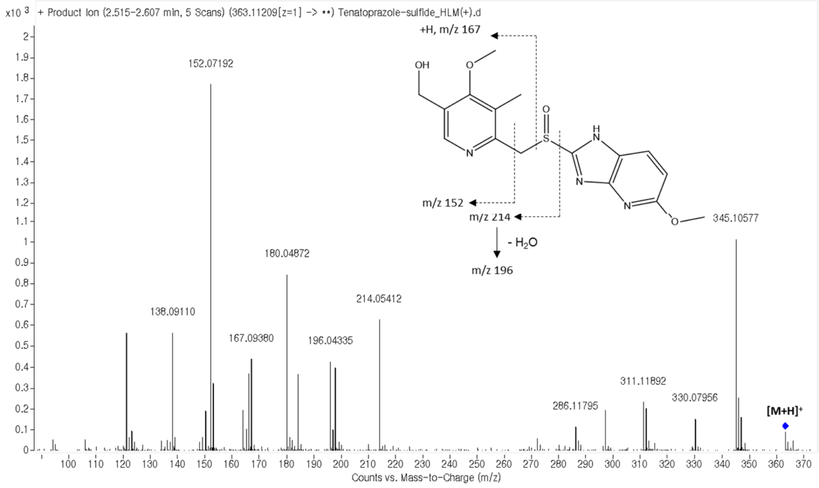

(H)

M4

- *m/z* 363

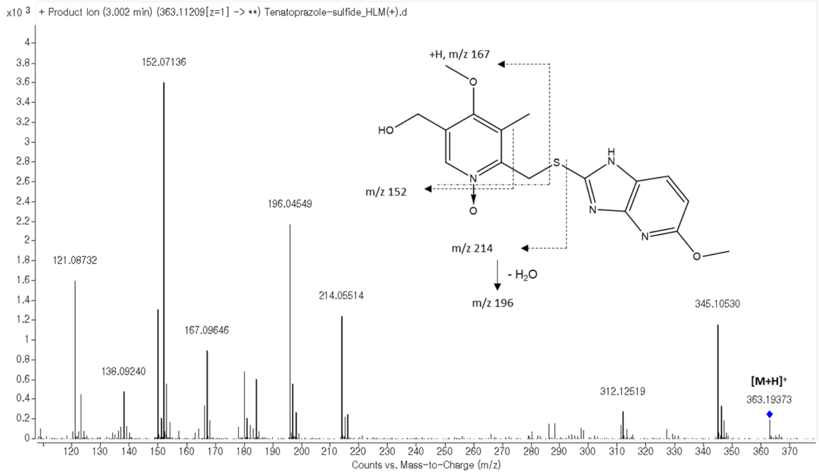

(I)

M5 & M6

-*m/z* 363

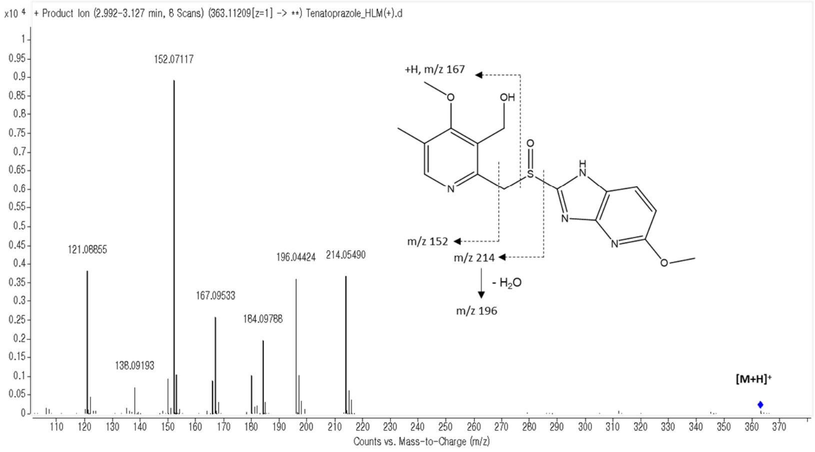

(J)

M7

- *m/z* 363

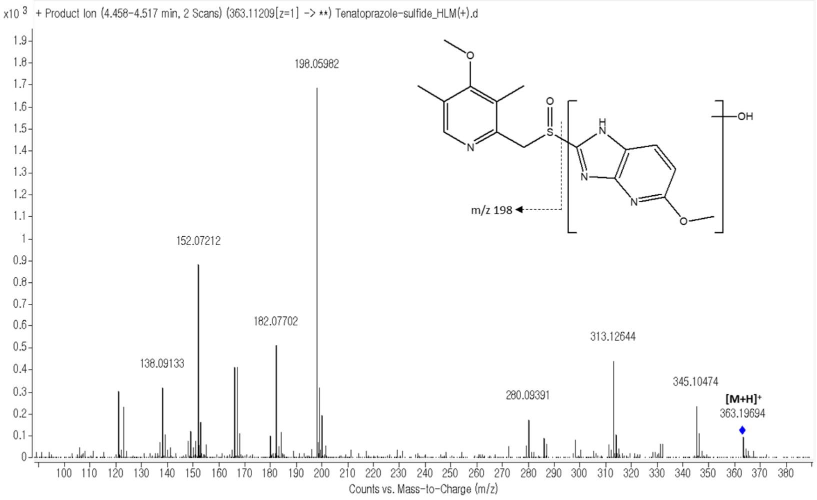

(K)

M8

- *m/z* 363

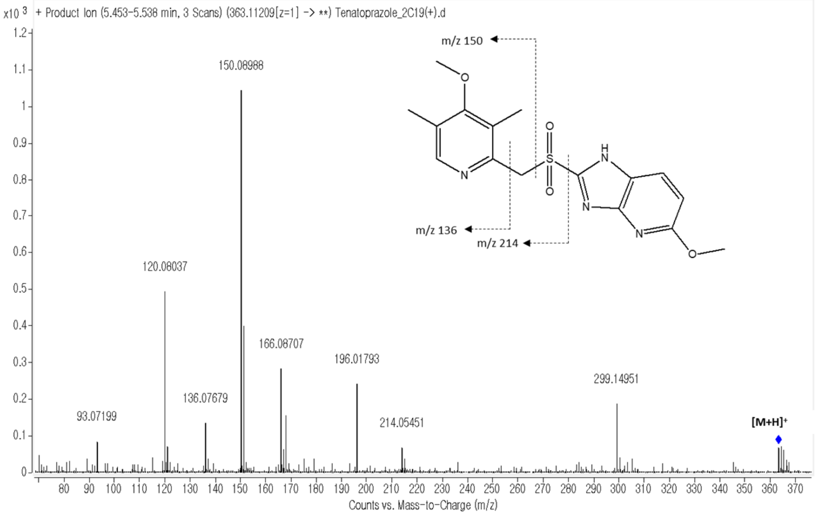

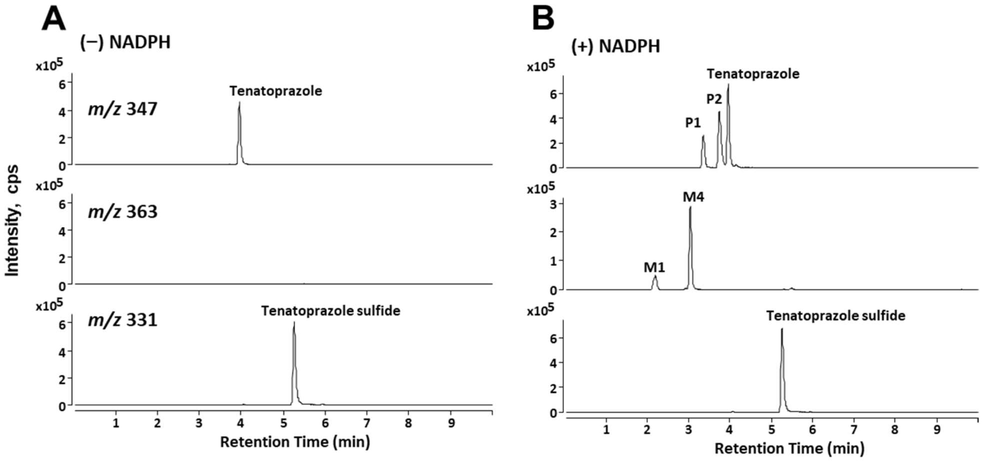

Figure S3. LC-QTOF-MS analyses of the metabolites from the tenatoprazole sulfide oxidation by CYP2C19. Extracted ion chromatograms were contracted from the incubation of tenatoprazole sulfide with CYP2C19 in the absence (**A**) and presence (**B**) of NADPH. The reaction mixture included 0.20 μM P450 and 0.10 mM tenatoprazole in 0.25 mL of potassium phosphate buffer (0.10 M, pH 7.4). The reaction was started by the addition of an NGS at 37 °C. After 30 min reaction, it was stopped by adding cold ethyl acetate (0.50 mL) and the upper layer was separated for LC-MS analysis.

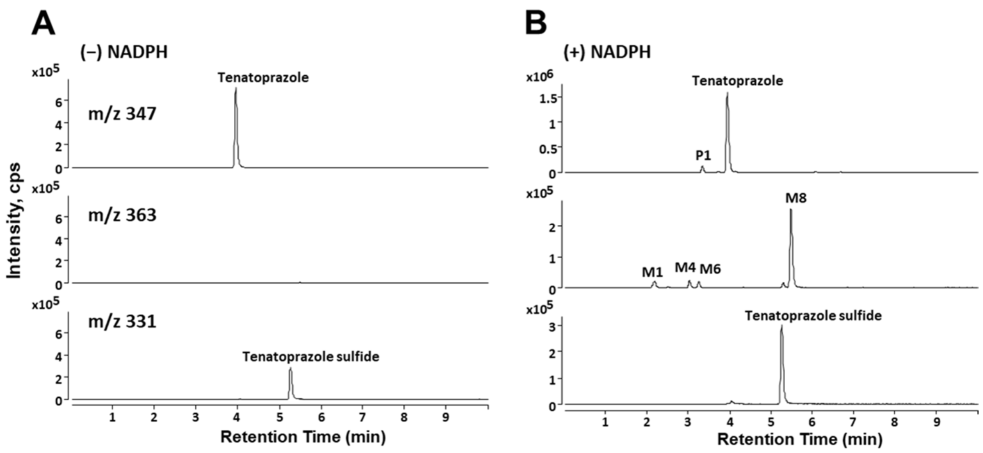

Figure S4. LC-QTOF-MS analyses of the metabolites from the tenatoprazole sulfide oxidation by CYP3A4. Extracted ion chromatograms were contracted from the incubation of tenatoprazole sulfide with CYP3A4 in the absence (**A**) and presence (**B**) of NADPH. The reaction mixture included 0.20 μM P450 and 0.10 mM tenatoprazole in 0.25 mL of potassium phosphate buffer (0.10 M, pH 7.4). The reaction was started by the addition of an NGS at 37 °C. After 30 min reaction, it was stopped by adding cold ethyl acetate (0.50 mL) and the upper layer was separated for LC-MS analysis.

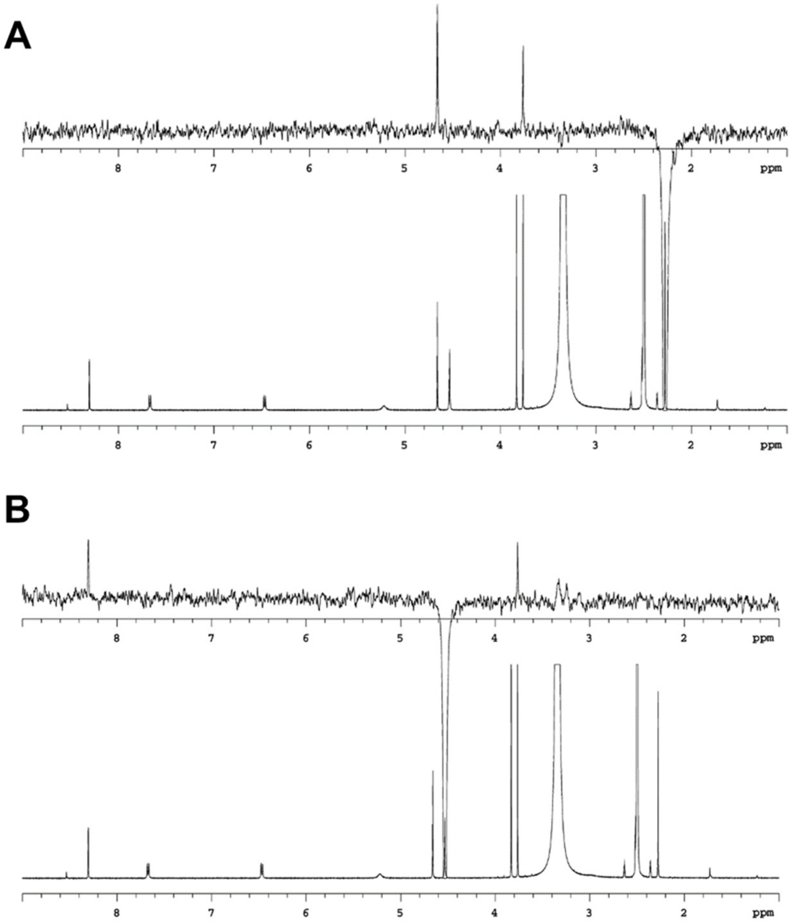

Figure S5. One-dimensional NOE spectrum of the metabolite (4). (A) One-dimensional NOE spectrum of the metabolite (4) after a selective irradiation at 2.279 ppm (3'-CH_3). Resulting NOE peaks corresponding to 4'-OCH_3 (3.764 ppm) and 2'-CH_2 (4.661 ppm) can be seen. (B) One-dimensional NOE spectrum of the metabolite (4) after a selective irradiation at 4.534 ppm (5'-CH_2). Resulting NOE peaks corresponding to H-6' (8.305 ppm) and 4'-OCH_3 (3.764 ppm) can be seen. Chemical structure of the metabolite (4) is 1'-*N*-oxy-5'-hydroxytenatoprazole sulfide (1′-*N*-oxy-5′-OH TNPS).

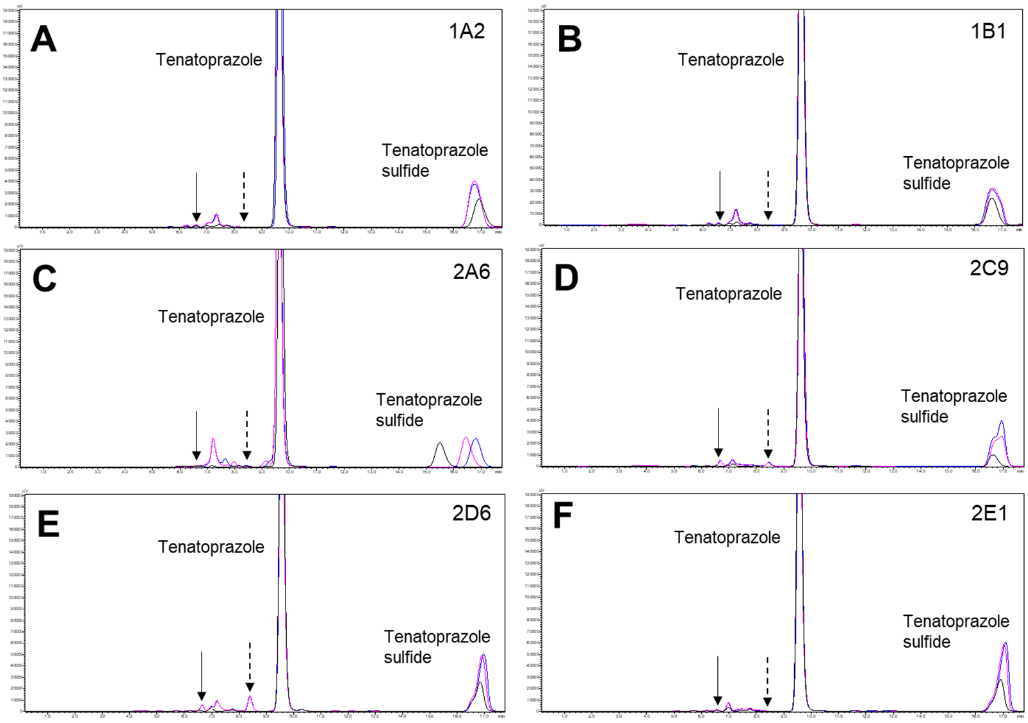

Figure S6. HPLC analyses of the products derived from the oxidation of tenatoprazole catalyzed by recombinant human P450s. The reaction mixture included 0.20 μM P450, 0.40 μM CPR, and 0.10 mM tenatoprazole in 0.25 mL of potassium phosphate buffer (0.10 M, pH 7.4). The reaction was started by adding an NGS at 37 °C for 60 min. (**A**) CYP1A2; (**B**) CYP1B1; (**C**) CYP2A6; (**D**) CYP2C9; (**E**) CYP2D6; (F) CYP2E1. The arrows with solid and broken lines indicate the retention times of 5′-OH tenatoprazole (6.6 min) and 5′-OH tenatoprazole sulfide (8.4 min), respectively.

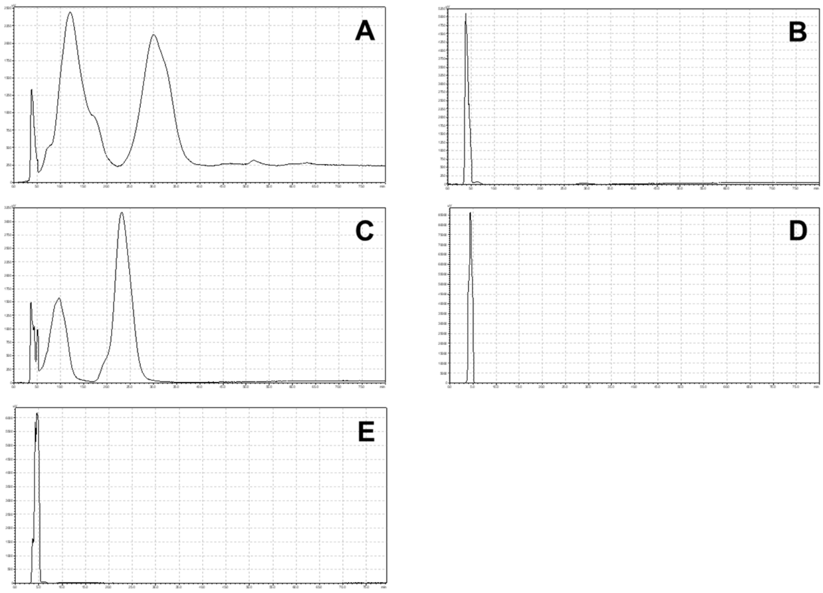

Figure S7. Chiral chromatography of metabolite mixture of tenatoprazole sulfide oxidation catalyzed by HLMs. (**A**) peak 1 (5′-OH tenatoprazole); (**B**) peak 2 (5′-OH tenatoprazole sulfide); (**C**) peak 3 (tenatoprazole); (**D**) peak 4 (1′-*N*-oxy-5′-OH tenatoprazole sulfide); (**E**) peak 6 (tenatoprazole sulfide).
